# Improving the Quality of Hospital Antibiotic Use: Impact on Multidrug-Resistant Bacterial Infections in Children

**DOI:** 10.3389/fphar.2020.00745

**Published:** 2020-05-15

**Authors:** Umberto Fanelli, Vincenzo Chiné, Marco Pappalardo, Pierpacifico Gismondi, Susanna Esposito

**Affiliations:** Department of Medicine and Surgery, Pediatric Clinic, Pietro Barilla Children's Hospital, University of Parma, Parma, Italy

**Keywords:** antimicrobial prescription, antimicrobial resistance, antimicrobial stewardship, children, pediatric infectious disease

## Abstract

Antimicrobial resistance (AMR) is considered a rapidly growing global public health emergency. Neonates and children are among patients for whom antibiotics are largely prescribed and for whom the risk of AMR development is high. The phenomenon of increasing AMR has led to the need to develop measures aimed at the rational and effective use of the available drugs also in children and antimicrobial stewardship (AS), which is one of the measures that in adults has showed the highest efficacy in reducing antibiotic abuse and misuse, appears as an attractive approach. The aim of this manuscript is to analyze the basic principles and strategies of pediatric AS. To this end, we searched in PubMed articles published in years 2000 to 2019 containing “antimicrobial resistance,” “antibiotic use,” “antimicrobial stewardship,” and “children” or “pediatric” as keywords. Our review showed that the balance between multi-resistant organisms and new antimicrobials is extremely precarious. The AS tools are the most important weapon at our disposal to stem the phenomenon. Careful monitoring of prescriptions, continuous training of prescribing physicians and collaboration with highly qualified multidisciplinary staff, creation of local and national guidelines, use of rapid diagnostic tests, technological means of support, and research activities by testing new broad-spectrum antibiotics are mandatory. However, all of these measures must be supported by adequate investment by national and international health organizations. Only by making AS daily practice, through the use of financial resources and dedicated staff, we can fight AMR to ensure safe and effective care for our young patients.

## Introduction

Antimicrobial resistance (AMR) is defined as the ability of a microorganism (bacterium, virus or parasite) to prevent an antimicrobial from acting against it (WHO, 2018a). Antibiotics not only can resolve current infections but also are responsible for the appearance of multidrug resistance (MDR); the term multidrug resistance (MDR) is used for bacteria that show drug resistance when three or more types of antimicrobial agents are used simultaneously in a clinical setting (D'Agata et al., 1997; Mitchell et al., 2014).

Historically, in the early stages of the antibiotic era, the rapid availability of new drugs made new therapies readily available to treat different infectious processes (between 1935 and 2003, 14 new classes of antibiotics were introduced). However, rapid antimicrobial development has a cost: AMR because of high prescription rates of antimicrobials (Doron and Davidson, 2011). At the same time, the discovery of new antibiotics has not kept pace with the increase in resistance in recent years. Hay et al. argue that the historical success rate of drug development is low, showing in their study that only one of five antimicrobials in phase 1 clinical trials will be approved (Hay et al., 2014). It is also estimated that fewer than half of the antibiotics currently under development have the potential to be used against MDR pathogens (Antibiotics Currently in Global Clinical Development).

AMR is considered a rapidly growing global public health emergency, both for epidemiological and economic reasons, to such an extent that the World Health Organization (WHO, 2019c) has published a relevant action plan. The emergence of multidrug-resistant bacterial pathogens is related to long hospital stays, increased patient mortality, and health care costs. For example, Roberts et al. evaluated that the health care costs of an antimicrobial-resistant infection to be between $18,588 and $29,069 per patient, with an excess hospital stay of 6.4 to 12.7 days and attributable mortality of 6.5% (Roberts et al., 2009).

Neonates and children are among patients for whom antibiotics are largely prescribed and for whom the risk of AMR development is high (Versporten et al., 2016). The phenomenon of increasing AMR has led to the need to develop measures aimed at the rational and effective use of the available drugs also in children and antimicrobial stewardship (AS), which is one of the measures that in adults has shown the highest efficacy in reducing antibiotic abuse and misuse, appears as an attractive approach (Doron and Davidson, 2011). Due to the limited available data in the pediatric population, the aim of this narrative review is to analyze the basic principles and strategies of pediatric AS. To this end, we searched in PubMed articles published in years 2000 to 2019 containing “antimicrobial resistance,” “antibiotic use,” “antimicrobial stewardship,” and “children” or “pediatric” as keywords.

## Overview on Antibiotic Resistance

Antibiotics are among the most widely prescribed medications among children in both hospitals and the community. According to data from ARPEC Study Group, the most frequently treated disease in children is bacterial infection of the lower respiratory tract (18.7%) (Versporten et al., 2016). In all regions except North America, beta-lactams account for ≥50% of all antibiotic prescriptions. A considerable amount of narrow-spectrum antibiotics is prescribed in Africa and Northern Europe (e.g., beta-lactamase-sensitive penicillins, 11.0% and 4.3%, respectively) and Australia (e.g., first generation cephalosporins, 9.6%). (Versporten et al., 2016). Moreover, it was found that the average percentage of children in hospital facilities receiving at least one antibiotic is between 33% and 78% (Grohskopf et al., 2005; Levy et al., 2012; Rutledge-Taylor et al., 2012). Although ARPEC Study Group collected data everywhere, only few information arrived from developing countries. On the other hand, many access antibiotics are unavailable in a majority of most health care facilities in low- and middle-income countries (Knowles et al., 2020).

Unfortunately, in both hospital and outpatient settings, the prescription of broad-spectrum antibiotics is often inappropriate or unnecessary (e.g., patients with viral infections or non-infectious diseases) in 50% of cases (Hecker et al., 2003). This practice is closely related to the increase in AMR (van de Voort et al., 2018) and other issues, including the increased incidence of *Clostridium difficile* infections (Baur et al., 2017). In 2019, Josephine et al. carried out a detailed analysis on the variability of prescriptions in certain hospitals (considering a population of children without comorbidities who had received antibiotics) by country, focus of the infection (particularly with respect to the respiratory tract) and type of antibiotic; they noted that differences in patient combination, diagnostic evaluation or hospital characteristics could not justify all the variability of antibiotic prescription, thus suggesting overuse (van de Maat et al., 2019). Interestingly, using the national health claims database, Kinoshita et al. retrospectively analyzed all oral antimicrobials dispensed from outpatient pharmacies in Japan to children under 15 years old from 2013 to 2016 by age, prefecture, type of antimicrobial, and year (Kinoshita et al., 2019). They showed that antimicrobials administered to pediatric patients in Japan did not decrease. Overall, antimicrobial prescriptions as well as prescriptions of cephalosporins, macrolides, and quinolones, were most prevalent in children ≤5 years old, highlighting the need of rigorous antimicrobial stewardship interventions.

The problem of AMR is also related to the large use of prophylaxis of infections in patients suffering from chronic diseases or undergoing surgery. The prophylactic use of antibiotics to prevent infections is controversial. Versporten et al. analyzed data on the prescription of prophylactic antibiotics from the ARPEC-PPS project (Versporten et al., 2013): most prescriptions were for medical prophylaxis (mostly in Western Europe, Australia, and North America), and only a quarter were for surgical prophylaxis instead. Regarding medical prophylaxis, oncological diseases were the most common underlying conditions in children after the neonatal period of life (Hufnagel et al., 2019).

## Antimicrobial Stewardship (AS)

AS has been conceptualized in many ways over the decades and intended as a set of coordinated interventions, programs, philosophy, and ethics. The term was coined in 1996 by two internists from the Emory University School of Medicine, namely, John McGowan and Dale Gerding, the latter a specialist in the *C. difficile* pathogen. They suggested large-scale, well-controlled studies on the use of antimicrobials through sophisticated epidemiological methods, typing of molecular biological organisms, and precise analysis of the mechanism of resistance in order to prevent and control this emerging problem (McGowan and Gerding, 1996). In 2001, Gerding et al. considered AS as the optimal selection, dosage, and duration of antimicrobial therapy that results in the best clinical outcome for the treatment or prevention of infection, with minimal toxicity to the patient and minimal impact on subsequent resistance (Gerding, 2001). The concept has been expanded over the years from a human perception to a more systemic one. In 2017, Dyar et al. reported AS as a coherent set of actions promoting the responsible use of antimicrobials, applicable at the individual, national, and global levels, with beneficial effects on human, animal, and environmental health (Dyar et al., 2017). In 2015, the Global Action Plan on Antimicrobial Resistance proposed the following five strategic points upon which AS should be based: 1) to improve awareness and understanding of AMR; 2) to strengthen knowledge through surveillance and research; 3) to reduce the incidence of infection; 4) to optimize the use of antimicrobial agents; 5) to develop an economic case for sustainable investment that takes into account the needs of all countries and increases investment in new medicines, diagnostic tools, vaccines, and other interventions (Resolution WHA 68-7, 2015).

In the creation of an AS program, the presence and close collaboration of a multidisciplinary team made up of specialized professionals, in both the hospital and the local area, according to their economic limits and available personnel is essential, whereas there are substantial differences between first- and third-level centers and among developing and industrialized countries. Ideally, the team should include a prescribing doctor, pharmacist, nurse, microbiologist, or laboratory technician. If available, an infectious disease specialist, a pharmacologist, and a nurse with experience in infection prevention and control are also recommended (Dellit et al., 2007).

For several years, the efforts of AS have focused on adult populations. The need to develop appropriate pediatric programs has been considered only in recent years, given the increasing use of antibiotics in children and the different antimicrobial resistance patterns of these patients compared to adults and the elderly. Over the past decade, multiple organizations, such as the Pediatric Infectious Diseases Society in 2010, the American Academy of Pediatrics in 2012, and the Infectious Disease Society of America (IDSA) in 2016, have emphasized the importance of AS in pediatrics and recognized pediatrics as a priority area for further research and activities (Principi and Esposito, 2016).

Among the reasons why it is so important to create a specific program for the pediatric patient is the heterogeneity in age and weight, which makes it difficult to apply a generic AS program, such as the adult one, given the lack of a standardized method to quantify the use of antibiotics in children. For example, one of the most widely used units of measurement for adults is the “defined daily dose” (DDD) introduced in the early 1980s by the WHO to measure the use of antibiotics in a standardized manner; however, the DDD is not easily applicable in pediatrics since it does not take into account the variability of dosage applied to weight (Di Pentima et al., 2011). Moreover, and not to be underestimated, there is the psychological aspect: the pediatrician often has to deal with the fears of parents, who are worried about the condition of their child, to which he/she could easily give in, causing an unnecessary antibiotic prescription. It follows that within an AS program, the educational aspect must be set in a specific way to respond to a very different type of need compared to that of a physician dealing with the adult patient. Recent data have clearly shown that antimicrobial resistance can be reduced by improving the use of antimicrobial agents and pediatric AS programs have a significant impact on the decrease of targeted and empiric antibiotic administration, health care costs, and antimicrobial resistance in both inpatient and outpatient settings (Chautrakarn et al., 2019; Donà et al., 2020). The most relevant AS methods are summarized below and are reported in **Table 1**.

**Table 1:** Main methods of antimicrobial stewardship (AS).

Programs of AS	Definition	Advantages	Disadvantages
PPs ON ANTIBIOTICS	Data collection on antimicrobial prescription and bacterial resistance at a given time.	Repeated PPSs represent a strategy to identify prescription trends over time, to assess the efficacy of AS and to address the problem of inappropriate antibiotic use.	A time-limited study cannot acquire data on treatment duration, clinical outcome or switching. Processing a PPS is more resource-intensive than simply collecting data on antimicrobial consumption. Tertiary care hospitals usually handle more complicated and serious cases, impacting on the type of antibiotic prescription. Participation in the studies is generally on a voluntary basis leading to an impairment of participation and data collection. Defined daily doses are not easily applicable in pediatrics since they do not take into account the variability of dosage applied to weight.
AWARE	A subdivision of antibiotics in three groups (Access, WAtch, REserve).	It facilitates the selection and monitoring of the correct antibiotic and to support AS.	Useful if applied together with other AS methods.
PRE-PRESCRIPTION AUTHORIZATION	“Restricted” indications for some antibiotics and their prescription by highly qualified personnel (e.g., doctors and pharmacists in the AS team).	It avoids abuse and/or inappropriate use of the chosen drugs. It reduces hospital costs.	Some clinicians may consider this method as a threat to their autonomy. Approval may be influenced by the variability of the authorizing subject. This method could lead to increased use of unauthorized antibiotics, sometimes undermining the objectives of the AS.
POST-PRESCRIPTION REVIEW	AS team members re-evaluate prescriptions and provide recommendations to physicians on the possibility to continue/modify/suspend. treatment, based on clinical-laboratory data.	Direct interaction and feedback with the prescriber. This method allows the authorizer to participate in the evolution of the therapy and to control it more efficiently.	It requires time and resources, which are not always available, from the AS team.
GUIDELINES AND DIAGNOSTIC ALGORITHMS	Facility treatment recommendations for common infection syndromes based on national or facility clinical guidelines, and on local susceptibility data, if available.	Lead to improved, standardized care for common infectious diseases. Help prescribers select initial therapy, improve antibiotic use, and decrease cost and length of stay.	Staff and adequate resources are not always available. For an optimal application, it is necessary to have a wide diffusion, including first-level centers and emergency departments with non-specialized pediatric personnel.
ANTIBIOTIC CYCLING	Antibiotics are withdrawn from intra-hospital use for a period—to limit the appearance of resistance—and are reintroduced afterward.	Theoretically, heterogeneity, as opposed to restriction of availability, is the most likely way to reduce the appearance of antibiotic resistance.	Practically, recent studies have also shown little usefulness in reducing antibiotic resistance.
RAPID DIAGNOSTIC TESTS	Tests that provide a result in advance of conventional methods used to identify the pathogenic microorganism.	They help in the choice of targeted therapy, in reducing hospital stays and costs as well as morbidity and mortality associated with infection.	They provide a qualitative and not a quantitative result. It is not possible to assess the severity of the disease, its monitoring, prognosis, and therapeutic efficacy. Testing is often expensive and/or requires advanced equipment not available in many facilities.
COMPUTERIZED PRESCRIBERS ORDER ENTRY	Process of entering and sending instructions from prescribing physicians, including medication orders, laboratory tests, and radiological examinations *via* a computer application rather than paper, fax, or telephone.	It reduces the error rate and improves patient safety. Clinical decision support tools can automatically verify drug interactions, drug allergies, and other potential problems. It can facilitate the rapid communication of drug orders and reports, saving time and improving efficiency.	Computerized prescribers order entry requires health care computer systems that are not always available in all health care facilities.
THERAPEUTIC DRUG MONITORING	Measurement of drug concentrations in biological fluids.	It assesses whether they are related to the patient's clinical condition or whether there is a need to change the dosage or intervals of administration.	It may be disadvantageous for clinicians not familiar with the constant use of these drugs. It may not be available in all facilities.

PPS, point-prevalence survey.

### Widely Adopted Strategies

#### Point Prevalence Survey

To promote effective interventions and thereby optimize the use of drugs, it is important to collect information on the current situation regarding the use of antibiotics in all countries. Data collection is necessary to provide a comprehensive and reliable picture of antibiotic use; however, it is often incomplete and fragmented. Hospitals are excellent places to assess antibiotic prescription modalities, given the high concentration of patients with different diseases: this often creates a high selection of bacteria due to the wide spectrum and quantity of antibiotics used, contributing to the development of antibiotic resistance. In most countries of the world, regular monitoring of antibiotic prescription data is not possible due to the high workload and lack of available resources. A viable alternative is to collect data at a specific point in time through the methodology of the point prevalence survey (PPS) (WHO, 2019c). The PPSs on the use of antibiotics in hospitals, therefore, aim to collect information on antimicrobial prescription and bacterial resistance at a given time. This allows participating hospitals to develop their antibiotic prescription practices and define plans to encourage their appropriate prescription. Through the quality of available data, it is possible to compare results within a single center and between different centers, leading to the availability of improvement plans at both the hospital and national levels (WHO, 2019b).

Historically, the first PPS projects have been conducted mainly on adults, providing minimal data on hospitalized children. For example, at the European level, the European Surveillance of Antimicrobial Consumption (ESAC) conducted three studies on the use of antibiotics in hospitals: in 2006 (ESAC-1), 2008 (ESAC-2), and 2010 (ESAC-3); however, the methodology used was not specifically designed for infants and children. With the objective of optimizing antimicrobial prescription in children, ARPEC conducted a PPS between 2011 and 2012 on the use of antimicrobials in hospitalized children, standardizing collected data based on age, sex, weight (at birth), basic diagnosis, antimicrobial agent, dose, and prescription indication (treatment of acute disease or medical/surgical prophylaxis). In addition, the following subdivisions according to the type of department were considered: neonatology, general pediatrics, specialists (cardiology, nephrology, oncohematology, neuromuscular, neurology, bronchopneumology, infectious diseases unit), and neonatal and pediatric intensive care (NICU and PICU) (De Luca et al., 2016). Through ARPEC, antimicrobial prescription data have been collected from over 200 hospitals and primary care sources, mainly in Europe. AMR models have been described for several pathogens, and existing antimicrobial prescription guidelines have been assembled. In the end, an educational tool was created to improve prescription in pediatrics. ARPEC's main legacy has been the promotion of successful collaborations between pediatricians in Europe and beyond: this led to GARPEC in 2014 (Global Antimicrobial Resistance, Prescribing, and Efficacy among Children Project), the global version of ARPEC, aimed not only at benchmarking AMR and prescribing worldwide but also at developing strategies to preserve antimicrobials in the face of increasing resistance (Vergnano et al., 2014).

PPSs have progressively included an increasing number of nations worldwide: ESAC-1, which has collected data from 20 hospitals in 20 different European countries, has developed into the ambitious project of a global PPS. A global PPS pilot project started in 2014, collecting data from infants, children, and adults in one day from 335 different hospitals in 53 countries, as follows: Europe (24 countries; 214 hospitals); Africa (five countries; 12 hospitals), Asia (16 countries; 57 hospitals), the Americas (six countries; 43 hospitals) and Oceania (two countries; nine hospitals). These analyses have been repeated annually since 2017, taking further into account seasonal and geographical variability (WHO, 2019b). However, PPS has certain disadvantages: 1) a time-limited study cannot acquire data on treatment duration, clinical outcome, or switching. Moreover, it is more likely to collect data on long-lasting therapies (Hsia et al., 2019); 2) processing a PPS is more resource-intensive than simply collecting data on antimicrobial consumption, as data are collected on individual patients (WHO, 2019a); 3) most institutions are actually tertiary care hospitals that usually handle more complicated and serious cases, and this could have a significant impact on the type of antibiotic prescription (De Luca et al., 2016); 4) participation in the studies is generally on a voluntary basis, and the researcher does not receive any payment. This factor could lead to an impairment of participation and data collection (Hufnagel et al., 2019).

In conclusion, repeated PPSs should be part of the pediatric management of antibiotics, as they represent a strategy to identify prescription trends over time, to assess the efficacy of AS and to address the problem of inappropriate antibiotic use, with particular regard to dosage. However, it has been shown that PPS alone is not enough to produce changes in clinical practice if they are not supported by other AS methods (Tersigni et al., 2019).

#### Aware

In March 2017, the WHO Essential Medicine List (EML) created three subcategories of antibiotics (Access, Watch, and Reserve) within the EML for children (EMLc) to facilitate the selection and monitoring of the correct antibiotic and to support AS. The first group is GROUP “Access” (green) includes antibiotics with broad spectrum activities that show a lower resistance potential than those contained in the other 2 groups. Antibiotics in this group should be used as first choice or as an empirical second choice treatment for the most common infections. The second is GROUP “WAtch” (yellow) that includes antibiotics with a broader spectrum but showing a higher resistance potential and should therefore be subject to priority local and national management and monitoring programs. They are used in first- or second-choice empirical treatment in a limited number of specific infectious diseases. The third is GROUP “REserve” (red) that includes antibiotics and classes of antibiotics that should be used for the treatment of confirmed or suspected infections due to multiresistant organisms, where all alternatives have failed or have not proven suitable (WHO, 2019a). In 2019, Hsia et al. suggested that PPS together with EMLs could be used as a valid and effective method to prescribe, monitor, and compare the use of antibiotics in children's hospitals (Hsia et al., 2019). The AWaRe Classification Database, which was recently expanded from essential medicine to 177 common antibiotics (WHO, 2020), can assist policy makers in adopting AWaRe as a tool to support setting performance targets and guide optimal use of antibiotics in countries. This tool can also be adopted by clinicians to monitor antibiotic use and implement surveillance activities at local level and inform the development of antibiotic treatment guidelines.

#### Prescription Authorization

Among the interventions of AS proposed for proper management of antibiotic therapy are pre-prescription authorization (PPA) and post-prescription feedback review (PPRF).

In the PPA approach, some antibiotics have restricted indications, and their prescription by clinicians must be previously approved by highly qualified personnel (e.g., doctors and pharmacists in the AS team) (Doron and Davidson, 2011). The advantages of this method are that it avoids abuse and/or inappropriate use of the chosen drugs while reducing hospital costs; one of the disadvantages is that some clinicians may consider this method as a threat to their autonomy; moreover, the approval may be influenced by the variability of the authorizing subject. Finally, this method could lead to increased use of unauthorized antibiotics, sometimes undermining the objectives of the AS (Philmon et al., 2006).

With regard to PPRF, AS team members re-evaluate prescriptions and provide recommendations to physicians regarding the possibility of continuing/modifying/suspending treatment based on clinical-laboratory data (Chung et al., 2013). Among the advantages are direct interaction and feedback with the prescriber; moreover, this method allows the authorizer to participate in the evolution of the therapy and to control it more efficiently and in accordance with the microbiological results (Doron and Davidson, 2011). At the same time, however, this requires time and resources, not always available, from the AS team.

Both of these approaches are valuable tools for AS enhancement, and over the years, several studies have tried to demonstrate which of the two is superior: Mehta et al. compared them in a study in a third-level hospital and found that the switch from PPA to PPRF was associated with a significant increase in both antimicrobials subject to authorization and the number of all antimicrobial agents included in the list of those who required authorization (Mehta et al., 2014). Among the few studies carried out in the pediatric field, Di Pentima et al. in 2011 introduced an AS methodology based mainly on PPA and PPRF in a third level pediatric hospital: a significant impact on the decrease of the use of antimicrobials was found, including both those under authorization and the rest, demonstrating the importance of these AS interventions on the quality of care of hospitalized children and the prevention of resistance (Di Pentima et al., 2011).

As for the method to be preferred, there are conflicting results in the literature. However, Chan et al. performed a further study in which they defined the impact of the PPA method on the use of vancomycin in a third level pediatric hospital and showed that, compared to the previous 3 years of use of the PPRF method, the addition of the second method did not significantly change the antibiotic administration (Chan et al., 2015). Two other studies conducted in pediatric hospitals in 2012 and 2017 reported a significant decrease in the general use of antibiotic therapy by implementing the PPRF method as part of the AS program (Cosgrove et al., 2012; Lighter-Fisher et al., 2017). It is currently believed that the PPRF method should be favored over the PPA if possible because it is considered superior (WHO, 2019a).

#### Guidelines and Diagnostic Algorithms

The inadequate prescription of antibiotics is often the result of poor education of clinicians, lack of appropriate guidelines, and lack of theoretical-practical training. Education is therefore considered a key point in AS programs and should include structured doctors and trainees, pharmacists, nurses, and students (Barlam et al., 2016). In fact, it is important that learning starts during the university period, through the implementation of AS-related concepts in textbooks, and continues during specialist training, taking advantage of daily clinical practice (Schrier et al., 2018).

Among the most recent methodologies is blended learning, a mix of online and face-to-face lessons, which is becoming increasingly popular among students (WHO, 2019a). Traditional educational techniques are moderately effective in increasing knowledge; conversely, interactive techniques improve prescriptive skills; these techniques include small group sessions, e-learning, educational awareness, periodic retrospective audits and feedback, and direct patient practice (Ohl and Luther, 2014). Guidelines on empirical antibiotic prescription and standard treatment lead to better treatment for the most common infectious diseases, help prescribers identify the right first-line therapy, and reduce costs and duration of stay (WHO, 2019a).

The usefulness of education and application of the guidelines has been demonstrated in many studies. Cisneros et al. demonstrated how the implementation of local guidelines on common infectious diseases combined with adequate training of clinicians in a tertiary center including pediatric patients improved the appropriateness of antibiotic treatment by 26.6% and was at the same time optimally accepted by clinicians (Cisneros et al., 2014). Recently, a study was carried out in a Swedish pediatric emergency room assessing adherence to national guidelines for acute otitis media and acute pharyngotonsillitis, combining educational awareness raising techniques and decision support. This easily implemented AS intervention was associated with a significant effect on the adequate prescription of antibiotics (Malmgren et al., 2019). Poole et al. conducted a retrospective cross-sectional study between 2009 and 2014 and found that there is a very high inappropriate antibiotic prescription rate among pediatric patients, thus emphasizing the need for expanding pediatric AS efforts to nonpediatric emergency rooms, particularly regarding avoidance of antibiotic prescribing for conditions for which antibiotics are not indicated, reducing macrolide use, and increasing first-line guideline-concordant prescribing. (Poole et al., 2019).

Regarding diagnostic algorithms, pediatric studies have demonstrated the utility of an algorithm based on a procalcitonin (PCT) cutoff value for guiding antibiotic administration in community-acquired pneumonia (CAP), showing that this approach can significantly decrease antibiotic prescription and antibiotic-related adverse events in pediatric patients with uncomplicated disease (Esposito et al., 2011).

Evidence-based guidelines and diagnostic algorithms appears a useful tool of AS. The guidelines are developed in specialized university centers, where both staff and adequate resources are available, or within Scientific Societies. However, for an optimal application, it is necessary to have a wide diffusion, including first-level centers and emergency departments with non-specialized pediatric personnel.

#### Antibiotic Cycling

A further proposed AS technique was antibiotic rotation: antibiotics are withdrawn from intrahospital use for a period of time—to limit the appearance of resistance—and are reintroduced afterwards. This is a structured way of introducing heterogeneity of antibiotics into prescribing practice, as heterogeneity, contrary to the restriction of availability, is the most likely way to reduce the appearance of antibiotic resistance (Robert, 2005).

The studies on this practice actually proved to be discordant: in 2002, Moss et al. performed a pilot research to define the safety effects of antibiotic rotation to decrease colonization and infection with antibiotic-resistant bacteria; the study was conducted in a 16-bed pediatric intensive care unit on critically ill children requiring antibiotic treatment. Three classes of antibiotics were rotated at 3-month intervals for 18 months. The broad-spectrum empirical antibiotic cycle was found to be safe and did not increase antibiotic resistance. The study concluded that antibiotic rotation could therefore be a safe and viable strategy to minimize antibiotic resistance in intensive care units (Moss et al., 2002). In the same year, Toltzis et al. examined whether monthly rotation of gentamicin, piperacillin-tazobactam, and ceftazidime could reduce colonization with Gram-negative resistant bacilli in a NICU. A total of 1,062 children were analyzed over a period of 1 year but in this study the rotation of parenteral antibiotics, according to the protocol applied, did not establish any impact in reducing the reservoir of resistant Gram-negative bacilli compared to the control group (Toltzis et al., 2002).Recent studies have also shown little usefulness in reducing antibiotic resistance (van Duijn et al., 2018).

#### Rapid Diagnostic Tests

Diagnostic uncertainty may lead to inappropriate antibiotic prescription. In addition, antibiotic therapy initiated on suspicion of a bacterial infectious disease is empirical until the microbiological tests results are available, which often requires prolonged periods of time. Empirical therapy is more complex in the pediatric field than in the adult field, considering the different etiology of infections according to age. A rapid diagnostic test (RDT) provides a result in advance of conventional methods used to identify pathogenic microorganisms (Kundu, 2018).

Two examples of RDTs used in pediatrics with an high impact in the reduction of antibiotic abuse are the one for the identification of *Streptococcus pyogenes* in pharyngitis (Chiappini et al., 2014) and the one for detection of influenza viruses (Esposito et al., 2003). Effectiveness of these tests have been reported in clinical studies, and the use of rapid test for *S. pyogenes* is included also in clinical guidelines.

RDTs help in the choice of targeted therapy, in reducing hospital stays and costs as well as morbidity and mortality associated with infection. However, an RDT should be easy to perform, should not require sophisticated machines, and its kits should be stable at extreme temperatures (Messacar et al., 2017; Reuter et al., 2019). In contrast, most available RDTs provide a qualitative and not a quantitative result; therefore, it is not possible to assess the severity of the disease, its monitoring, prognosis, and therapeutic efficacy (Kundu, 2018). Finally, testing is often expensive and/or requires advanced equipment not available in many facilities (WHO, 2019a).

### Potential Alternatives

#### Computerized Prescribers Order Entry

Computerized Prescribers Order Entry (CPOE) refers to the process of entering and sending instructions from prescribing physicians, including medication orders, laboratory tests, and radiological examinations *via* a computer application rather than paper, fax, or telephone.

Children are particularly vulnerable to medication errors for multiple reasons: use of doses based on weight or body surface area, increased sensitivity to medication errors, and variable ability to metabolize and excrete drugs. CPOE can reduce the error rate and improve patient safety. In addition, CPOE technology often includes clinical decision support tools that can automatically verify drug interactions, drug allergies, and other potential problems. By enabling suppliers to send orders electronically, CPOE can facilitate the rapid communication of drug orders and laboratory and radiology reports to pharmacies, laboratories, and radiology facilities, saving time and improving efficiency. Finally, CPOE can facilitate the reimbursement of expenses if integrated with a practical electronic management system (WHO, 2018b). The study by Chaparro et al. carried out from 2008 to the end of 2010 found that an average pediatric CPOE system was able to identify 62% of therapeutic errors compared to 44.3% in the adult system, while demonstrating the validity of this method and the need to develop specific and validated systems in children (Chaparro et al., 2017). Unfortunately, the CPOE requires health care computer systems that are not always available in all health care facilities (WHO, 2019a).

#### Therapeutic Drug Monitoring

One of the methods to optimize the use of antimicrobials and improve clinical outcome is therapeutic drug monitoring (TDM) (Roberts et al., 2012). TDM is defined as the measurement of drug concentrations in biological fluids to assess whether they are related to the patient's clinical condition or whether there is a need to change the dosage or intervals of administration. The criteria for monitoring drugs in pediatric age are similar to those for adults, but other factors as the physiological and biochemical changes related to age (particularly during the first year of life) resulting in different pharmacokinetic and clinical pharmacodynamic parameters must be considered. When administering drugs to children, age-related differences in drug absorption, distribution, metabolism, and clearance must be taken into account to optimize drug efficacy and avoid toxicity (Soldin and Soldin, 2002; Downes et al., 2014). Modeling of the pharmacokinetic/pharmacodynamic (PK/PD) characteristics of antibiotics can support the optimization of dosing regimens. The three clinical applications of these models applied to antibiotics: 1) dosing evaluation of old antibiotics, 2) setting clinical breakpoints, and 3) dosing individualization using TDM (de Velde et al., 2018).

TDM is indicated for concentration-dependent antibiotics, e.g., vancomycin and aminoglycosides, when used for at least 3 days (WHO, 2019a) and often involves the pharmacist who can choose the initial dose and make changes based on blood levels using electronic calculators. Among the critical issues, TDM may be disadvantageous for clinicians not familiar with the constant use of these drugs (Doron and Davidson, 2011). Moreover, this type of monitoring may also not be available in all facilities (WHO, 2019a).

## Antimicrobial Stewardship (AS) in Outpatient Setting: A Priority for Reduction of Antibiotic Abuse

Although all the methods mentioned above can be used in outpatient setting, AS interventions on outpatients are less frequent. Indeed, the few studies conducted show that AS can be effective in reducing the misuse of antibiotics even in outpatient clinics, but with less relevant results than those conducted in a hospital environment (Principi and Esposito, 2016). Among such studies, one of the most important was conducted in Sweden, incorporating adults and children visited on an outpatient basis. The consumption of antibiotics by children between 0 and 6 years of age has decreased by 54.6% since 2000 (from 746 to 339 prescriptions per 1,000 children and year), showing great variability of prescription between the Swedish counties investigated. In this age group, the following penicillins beta-lactams were the most commonly prescribed antibiotics: penicillin V, amoxicillin, and flucloxacillin (O'Donnell and Guarascio, 2017; Sweders Swarm, 2017).

In a recent article, Zetts et al. reported that in the United States the highest prescription rate of antibiotics occurs on an outpatient basis, and approximately 30% is inappropriate (Szymczak et al., 2014). In addition to AMR, this also increases the likelihood of adverse drug reactions and the development of *Clostridium difficile* infections. Antibiotics were prescribed for acute respiratory tract infections, including rhinosinusitis, acute otitis media, pharyngitis, viral upper respiratory tract infection, bronchitis and bronchiolitis, asthma and allergy, influenza, and bacterial or viral pneumonia. The highest prescription rate was reported among pediatric patients under 2 years of age, and the most prescribed antibiotics were penicillins, macrolides, and cephalosporins. The study lists many factors that lead the doctor to prescribe antibiotics incorrectly, some of them unrelated to the clinical aspect (e.g., pressure from parents who expect tangible actions to deal with the child's illness) (Szymczak et al., 2014). Another study confirmed that the figure of the parent can be a major problem for antibiotic abuse: 24 pediatricians interviewed identified parental pressure as a serious obstacle to the more prudent use of antibiotics (Zetts et al., 2018). Respondents reported that they sometimes succumbed to parental pressure for social reasons. The culture of expectation on the part of parents is therefore a very topical problem that pediatricians often face when prescribing antibiotics (Szymczak et al., 2014). Physicians operating in private practice may be particularly sensitive to losing patients to their competitors if the parents are not satisfied with a non-prescription approach. In clinics, doctors have limited time to make a diagnosis after assessing patients with non-specific symptoms to begin the most appropriate treatment.

Decision fatigue resulting from the doctor's workload can also contribute to inappropriate antibiotic prescription: it often takes less time to prescribe an antibiotic than to provide a lengthy explanation as to why it is not necessary (Zetts et al., 2018).

In addition, specific diagnostic tests to discriminate whether the infection is of viral or bacterial origin are not always available (Centers for Disease Control and Prevention, 2016). In 2016, the Centers for Disease Control and Prevention (CDC) published the basic principles of AS for extra-hospital managed patients. These include the need for constant efforts to show the problems related to AMR (e.g., through posters in waiting rooms). Monitoring and reporting of the antibiotic prescription are important, requiring, for example, a written justification for the use of non-recommended antibiotics and the encouragement of continuous training for clinicians to minimize error. Recently, building on key elements published by the CDC, it has been reaffirmed how effective interventions of AS can protect children and optimize clinical outcomes not only in hospital, but also in outpatient settings (Kilgore and Smith, 2019).

## Conclusions

The problem of AMR in both hospital and community settings represents a global emergency in terms of individual health and health care costs. The balance between multi-resistant organisms and new antimicrobials is extremely precarious. The AS tools are the most important weapon at our disposal to stem the phenomenon. The AS strategies conducted on adult patients have had a positive impact; only recently have they been undertaken in the pediatric field, where targeted interventions are needed, considering the heterogeneity in age and weight of patients. Careful monitoring of prescriptions, continuous training of prescribing physicians, and collaboration with highly qualified staff, creation of local and national guidelines, use of RDTs, technological means of support, and research activities by testing new broad-spectrum antibiotics are mandatory. However, all of these measures must be supported by adequate investment by national and international health organizations. Only by making AS daily practice, through the use of financial resources and dedicated staff, we can fight AMR to ensure safe and effective care for our young patients.

## Author Contributions

UF, VC, and MP performed the literature review and wrote the first draft of the manuscript. PG and SE critically revised the text and made substantial scientific contributions. All authors approved the final version of the manuscript.

## Conflict of Interest

The authors declare that the research was conducted in the absence of any commercial or financial relationships that could be construed as a potential conflict of interest.
